# Variants in *ABCG8* and *TRAF3* genes confer risk for gallstone disease in admixed Latinos with Mapuche Native American ancestry

**DOI:** 10.1038/s41598-018-35852-z

**Published:** 2019-01-28

**Authors:** Bernabé I. Bustos, Eduardo Pérez-Palma, Stephan Buch, Lorena Azócar, Eleodoro Riveras, Giorgia D. Ugarte, Mohammad Toliat, Peter Nürnberg, Wolfgang Lieb, Andre Franke, Sebastian Hinz, Greta Burmeister, Witigo von Schönfels, Clemens Schafmayer, Henry Völzke, Uwe Völker, Georg Homuth, Markus M. Lerch, José Luis Santos, Klaus Puschel, Claudia Bambs, Juan Carlos Roa, Rodrigo A. Gutiérrez, Jochen Hampe, Giancarlo V. De Ferrari, Juan Francisco Miquel

**Affiliations:** 1 Institute of Biomedical Sciences, Faculty of Medicine and Faculty of Life Sciences, Universidad Andrés Bello, Santiago, Chile; 2 Medical Department I, University Hospital Dresden, TU Dresden, Dresden, Germany; 3 Departmento de Gastroenterología, Faculty of Medicine, Pontificia Universidad Católica de Chile, Santiago, Chile; 4 Cologne Center for Genomics, University of Cologne, Cologne, Germany; 5 Center for Molecular Medicine Cologne (CMMC), University of Cologne, Cologne, Germany; 6 Cologne Excellence Cluster on Cellular Stress Responses in Aging-Associated Diseases (CECAD), University of Cologne, Cologne, Germany; 7 Institute of Epidemiology and Biobank PopGen, Christian-Albrechts-University of Kiel, Kiel, Germany; 8 Institute of Clinical Molecular Biology, Christian-Albrechts-University of Kiel, Kiel, Germany; 9 Department of Visceral and Thoracic Surgery, University Hospital Schleswig Holstein, Kiel, Germany; 10 Institute for Community Medicine, University Medicine Greifswald, Greifswald, Germany; 11 Interfaculty Institute for Genetics and Functional Genomics, University Medicine Greifswald, Greifswald, Germany; 12 Department of Nutrition, Diabetes and Metabolism, School of Medicine, Pontificia Universidad Católica de Chile, Santiago, Chile; 13 Department of Familiar Medicine, Faculty of Medicine, Pontificia Universidad Católica de Chile, Santiago, Chile; 14 Department of Public Health, Faculty of Medicine, Pontificia Universidad Católica de Chile, Santiago, Chile; 15 Departamento de Anatomía Patológica, Facultad de Medicina, Pontificia Universidad Católica de Chile, Santiago, Chile; 16 Department of Molecular Genetics and Microbiology, Faculty of Biological Sciences, Pontificia Universidad Católica de Chile, Santiago, Chile; 17 FONDAP Center for Genome Regulation (CGR), Santiago, Chile

## Abstract

Latin Americans and Chilean Amerindians have the highest prevalence of gallstone disease (GSD) and gallbladder cancer (GBC) in the world. A handful of loci have been associated with GSD in populations of predominantly European ancestry, however, they only explain a small portion of the genetic component of the disease. Here, we performed a genome-wide association study (GWAS) for GSD in 1,095 admixed Chilean Latinos with Mapuche Native American ancestry. Disease status was assessed by cholecystectomy or abdominal ultrasonography. Top-10 candidate variants surpassing the suggestive cutoff of P < 1 × 10^−5^ in the discovery cohort were genotyped in an independent replication sample composed of 1,643 individuals. Variants with positive replication were further examined in two European GSD populations and a Chilean GBC cohort. We consistently replicated the association of *ABCG8* gene with GSD (rs11887534, P = 3.24 × 10^−8^, OR = 1.74) and identified *TRAF3* (rs12882491, P = 1.11 × 10^−7^, OR = 1.40) as a novel candidate gene for the disease in admixed Chilean Latinos. *ABCG8* and *TRAF3* variants also conferred risk to GBC. Gene expression analyses indicated that TRAF3 was significantly decreased in gallbladder (P = 0.015) and duodenal mucosa (P = 0.001) of GSD individuals compared to healthy controls, where according to GTEx data in the small intestine, the presence of the risk allele contributes to the observed effect. We conclude that *ABCG8* and *TRAF3* genes are associated with GSD and GBC in admixed Latinos and that decreased TRAF3 levels could enhance gallbladder inflammation as is observed in GSD and GSD-associated GBC.

## Introduction

Gallstone disease (GSD) is a complex gastrointestinal disorder defined by the presence of gallstones in the gallbladder, most of the time made of cholesterol compounds^1^. The presence of gallstones is common in the world population (~10–20% of presence in adults) and although their appearance can remain silent throughout life, more than 20% of the patients develop symptoms that include intense abdominal pain, jaundice, fever, nausea and vomiting, requiring medical intervention^2^. Main complications of GSD are acute cholecystitis, acute pancreatitis and bile duct obstruction^3^, which are generally treated by surgical removal of the gallbladder (cholecystectomy). In Chile, these procedures account for more than 40,000 interventions each year^4^, with a net cost of more than US $25 million to the public healthcare system. Additionally, GSD is the main risk factor for gallbladder cancer (GBC), a disease that presents a high mortality rate in Chile, with 38.2 deaths per 100,000 inhabitants^5^. In the majority of cases, GBC is an adenocarcinoma characterized by high lethality due to late diagnosis and the ineffectiveness of chemotherapy/radiotherapy. With the removal of the gallbladder and its stones, GBC risk is considerably reduced^6^.

Epidemiological studies have shown that ethnicity plays a major role in the prevalence of GSD. Asian and African countries have the lowest prevalence of the disease (<10%), followed by European (~20%) and American (>40%) countries^2^. Notably, the prevalence in adult Chilean Mapuche Amerindians is estimated to be 49.4% and 12.3% for women and men, respectively^7^. Likewise, adult Chilean women and men from the general population, resulting from the admixture of Europeans (mainly from Spain) and indigenous Amerindians (mainly Mapuches), have an estimated prevalence of 36.7% and 13.1%, respectively^7^. Studies in families and case-controls populations have shown that GSD has a strong genetic component^8,9^. The most important genetic risk factor is the ATP-binding cassette, sub-family G (WHITE), member 8, *ABCG8* gene^10^, an hepatic sterolin transporter where the associated polymorphism (p.D19H, rs11887534) is linked to a gain-of-function responsible for the hyper secretion of cholesterol-saturated bile^11^. Another important risk factor is the UDP glucuronosyl-transferase 1 family, polypeptide A1, *UGT1A1* gene, which has been significantly associated to GSD only in men. Both signals have been replicated in different populations across the world, including admixed Chileans^10,12,13^, yet they only explain a small portion of the population attributable risk of the disease (*ABCG8* PAR = 11.2%, *UGT1A1* PAR = 9.9%, combined PAR = 21.2%)^14^. Recently, a meta-analysis involving 8,720 GSD cases and 55,152 controls identified four additional risk factors, including the Transmembrane 4 L six family member 4, *TM4SF4* gene (rs9843304), the Sulfotransferase family 2 A member 1 *SULT2A1* gene (rs2547231), the glucokinase regulator, *GCKR* gene (rs1260326) and the cytochrome P450 family 7 subfamily A member 1, *CYP7A1* gene (rs6471717)^15^. However, none of these novel risk factors have been studied in the Chilean population.

Here we report the results of the first large GWAS for a prevalent complex disease in the admixed Chilean population. We hypothesized that a high-density GWAS for GSD in admixed Latinos could identify population specific variants, define the GSD genetic landscape and reveal novel pathological mechanisms.

## Results

### Discovery GWAS for GSD in an admixed Chilean population

The Discovery GWAS stage involved 1,235 admixed Chileans Latinos with Mapuche Native American Ancestry genotyped using the Affymetrix Axiom® Genome-Wide LAT 1 World Array 4 (see complete pipeline in Supplementary Fig. 1). After genotype calling and quality controls, 1,095 individuals (529 GSD cases and 566 controls) and 677,835 SNPs remained for analysis (Table 1). We imputed genotypes in these individuals using the 1000 Genomes Project Phase 3 reference panel^16^ and achieved a total number of 9,433,911 SNPs and Indels. We tested for genetic association with GSD by performing a logistic regression analysis adjusted by age (in years; cases = 51.32 ± 10.67, controls = 49.87 ± 9.57, one-way ANOVA P = 0.018), since other known associated covariates such as sex (women%; cases = 92.43%, controls = 92.57%, Z score test P = 0.928), body mass index (BMI; cases = 29.44 ± 4.28, controls = 29.66 ± 3.94, one-way ANOVA P = 0.376) and native American ancestry proportion (cases = 46.3% ± 7.0%, controls = 45.5% ± 7.5%, one-way ANOVA P = 0.069), did not differ significantly between cases and controls.

**Table 1:** Clinical characteristics of patients in the Discovery GWAS and independent replication populations.

Variable	Discovery^a^		Replication^a^		Chile GBC	Secondary replication populations^b^			
	Stage 1 (n = 1,095)		Stage 2 (n = 1,643)			Germany POPGEN-KIEL (n = 1,938)		Germany SHIP-Greifswald (n = 4,154)	
	Cases (n = 529)	Controls (n = 566)	Cases (n = 626)	Controls (n = 1,017)	Cases (n = 397)	Cases (n = 1,027)	Controls (n = 911)	Cases (n = 882)	Controls (n = 3,272)
Age (years)^c^	51.32 ± 10.67	49.87 ± 9.57	59.57 ± 12.47	48.26 ± 12.19	62.66 ± 12.85	45.54 ± 12.25	59.43 ± 12.79	61.02 ± 13.10	47.12 ± 15.91
Sex (% women)	92.43	92.57	84.82	63.12	100	60.56	42.04	65.53	46.97
BMI^c^	29.44 ± 4.28	29.66 ± 3.94	30.74 ± 5.92	28.61 ± 5.38	N.A.	27.61 ± 5.29	26.46 ± 4.13	27.95 ± 3.89	26.67 ± 4.51
T2D (% affected)	0	0	40.73	14.36	N.A.	N.A.	N.A.	17.4	6.4

^a^Chilean Discovery and Replication populations are described in Materials and Methods.

^b^Secondary replication populations correspond to analyses to measure TRAF3 association in different populations where not all necessary data (covariates) where available for adjustment.

^c^All quantitative measures are shown as average and its standard deviation; T2D, Type 2 diabetes; N.A., not available.

**Figure 1:**
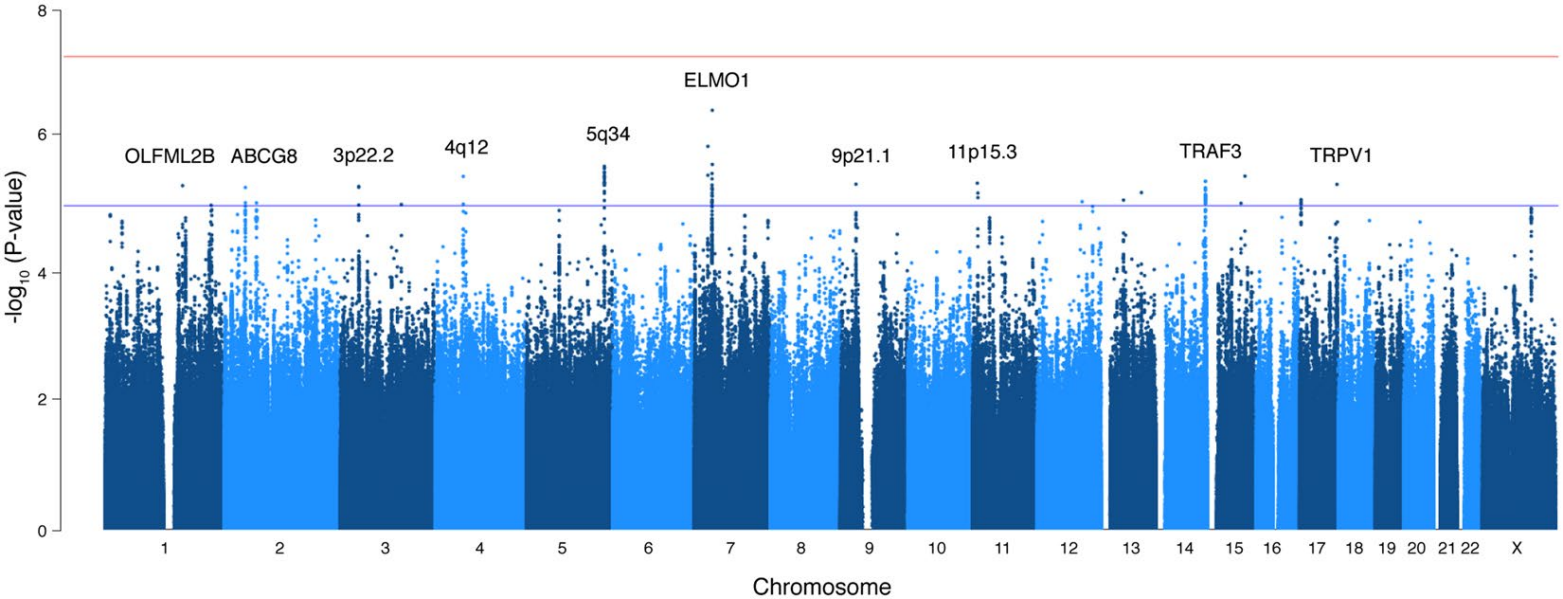
Genome-wide association results for GSD in admixed Chileans in the discovery stage. Manhattan plot depicting the association P values for all good quality variants. Red line shows genome-wide significance threshold (P < 5 × 10^−8^). Top-ten candidate variants surpassing the suggestive genome-wide significance threshold (blue line, P < 1 × 10^−5^) are shown, which were taken for further replication.

Genome-wide association results are presented in Fig. 1 and consider a genomic inflation factor λ = 1.02 (Supplementary Fig. 2). While we did not detect genome-wide significant associations, several suggestive signals were observed (P < 1 × 10^−5^). The top-10 most significant loci were: *ELMO1* (rs4446645, P = 3.36 × 10^−7^), 5q34 locus (rs10463138, P = 2.47 × 10^−6^), 4q12 locus (rs74537816, P = 3.54 × 10^−6^), *TRAF3* (rs368550004, P = 3.92 × 10^−6^), 9p21.1 locus (rs4879592, P = 4.66 × 10^−6^), *OLFML2B* (rs10918361, P = 4.91 × 10^−6^), 3p22.2 locus (rs73827633, P = 5.06 × 10^−6^), *ABCG8* (rs11887534, P = 5.24 × 10^−6^), 11p15.3 locus (rs147367002, P = 7.63 × 10^−6^) and *TRPV1* (rs7223530, P = 8.07 × 10^−6^) (Table 2). Aside from *ABCG8*, none of the previous GWAS signals associated with GSD reported in a recent large meta-analysis of individuals with European ancestry^15^ reached suggestive genome-wide significance (Supplementary Table 1).

**Table 2:** Top-10 candidate variants associated with GSD in admixed Chileans in the discovery and replication stages.

SNP	Locus	Chr	SNP ID	Genotyping^b^	RA	RAF	Discovery			Replication			Combined^a^		
							HWE P value^c^	P value	OR (95% CI)	HWE P value^c^	P value	OR (95% CI)	Meta P value^d^	I^2e^	OR (95% CI)
SNP 1	*ELMO1*	7	rs4446645	Imputation	A	0.66	0.44	3.36 × 10^-7^	1.55 (1.31-1.84)	0.51	0.72	1.03 (0.87–1.23)	—	—	—
SNP 2	5q34	5	rs10463138	Imputation	A	0.52	0.44	2.47 × 10^−6^	1.50 (1.27–1.78)	0.45	0.43	0.94 (0.79–1.10)	—	—	—
SNP 3	4q12^f^	4	rs74537816	Imputation	T	0.95	0.46	3.54 × 10^–6^	2.29 (1.61–3.25)	—	—	—	—	—	—
		4	rs1824387	Chip	A	0.96	0.42	3.95 × 10^-5^	2.05 (1.46–2.90)	2 × 10^-40^	0.55	1.09 (0.82–1.45)	—	—	—
SNP 4	*TRAF3* ^f^	14	rs368550004	Imputation	G	0.69	0.34	3.92 × 10^−6^	1.52 (1.27–1.81)	0.13	0.009	1.26 (1.06–1.50)	—	—	—
		14	rs12882491	Imputation	C	0.69	0.38	5.28 × 10^−6^	1.50 (1.26–1.78)	0.13	0.003	1.30 (1.09–1.54)	1.11 × 10^-7^	24.68	1.40 (1.20–1.60)
SNP 5	9p21.1	9	rs4879592	Imputation	C	0.86	0.63	4.66 × 10^−6^	1.66 (1.34–2.06)	0.46	0.35	1.11 (0.89–1.40)	—	—	—
SNP 6	*OLFML2B*	1	rs10918361	Chip	G	0.43	0.40	4.91 × 10^-6^	1.50 (1.26–1.78)	0.99	0.09	0.86 (0.72–1.02)	—	—	—
SNP 7	3p22.2	3	rs73827633	Imputation	T	0.98	0.64	5.06 × 10^-6^	2.98 (1.86–4.76)	0.11	0.73	0.93 (0.63–1.39)	—	—	—
SNP 8	*ABCG8*	2	rs11887534	Chip	C	0.14	1	5.24 × 10^-6^	1.88 (1.43–2.47)	0.54	0.001	1.59 (1.20–2.11)	3.24 × 10^-8^	0	1.74 (1.45–2.02)
SNP 9	11p15.3^f^	11	rs147367002	Imputation	A	0.14	1	7.63 × 10^-6^	1.94 (1.45–2.59)	—	—	—	—	—	—
		11	rs16908929	Imputation	A	0.15	0.17	4.30 × 10^-5^	1.76 (1.34–2.32)	0.004	0.47	1.09 (0.86–1.39)	—	—	—
SNP 10	*TRPV1*	17	rs7223530	Imputation	G	0.77	0.33	8.07 × 10^−6^	1.55 (1.28–1.88)	0.39	0.27	0.90 (0.74–1.09)	—	—	—

^a^Combined analysis correspond only to the variants surpassing Bonferroni correction (P < 0.005) at replication stage. ^b^Genotyping method for the candidate variant in the discovery population. ^c^Hardy-Weinberg equilibrium p-values calculated in control samples. ^d^Meta-analysis *P* value correspond to Fixed effect estimates. ^e^I^2^ indicates between-study heterogeneity. ^f^The leading variants selected for the candidates were not genotyped by TaqMan in the replication samples due technical issues and were replaced by adjacent SNPs in high LD: rs1824387 for SNP 3, r^2^ = 0.88; and rs16908929, r^2^ = 0.88; respectively. ^e^For *TRAF3* we genotyped the leading variant rs368550004 corresponding to an Indel and the best SNP option in high LD rs12882491, r^2^ = 1.0. RA: risk allele; RAF: risk allele frequency; OR: odds ratio; CI: confidence interval.

### Association of *ABCG8* and *TRAF3* variants with GSD in an independent Chilean population

**Figure 2:**
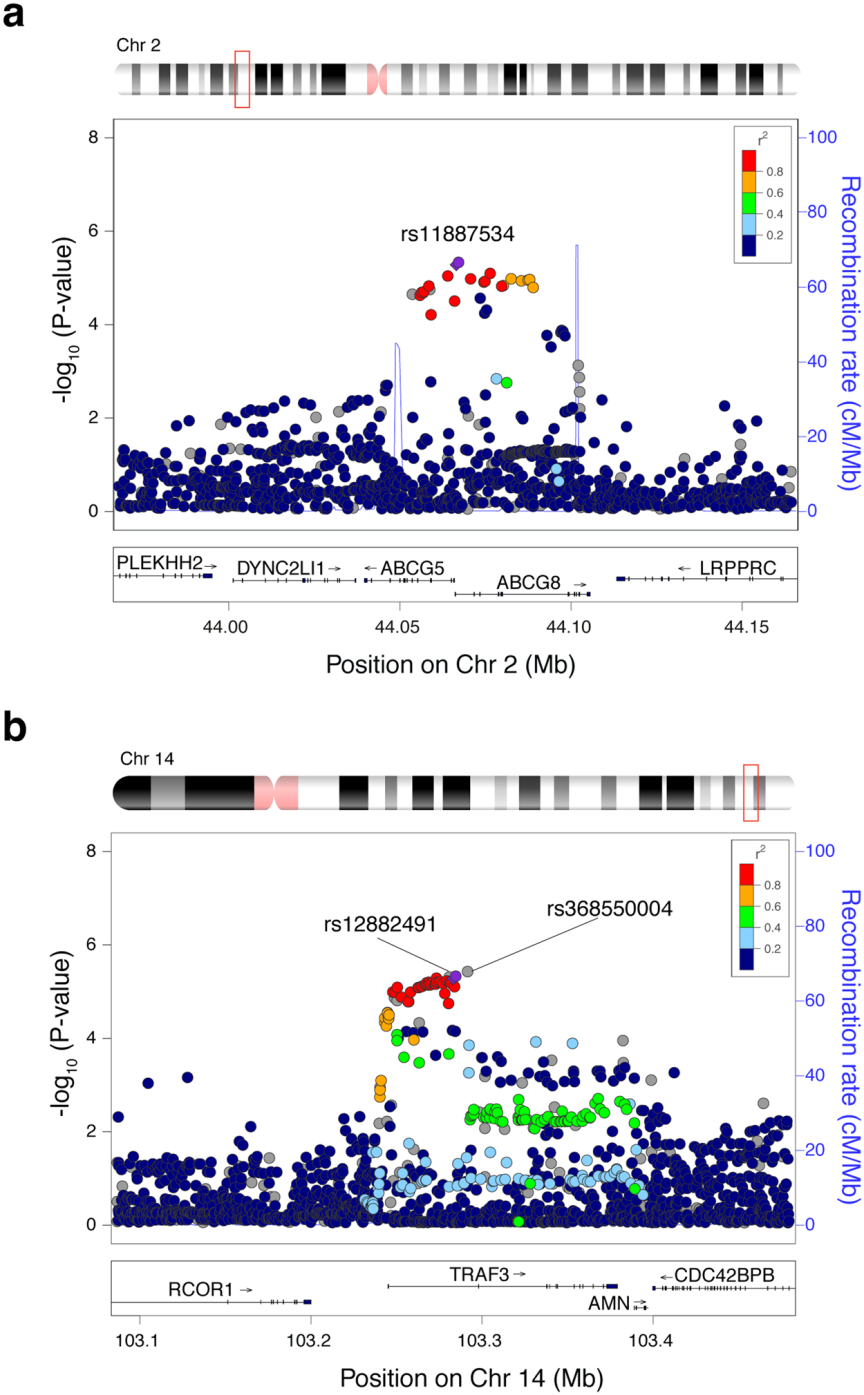
Regional association plots for the *ABCG8* and *TRAF3* signals in the GWAS discovery stage. Locus zoom for *ABCG8* (**a**) and *TRAF3* (**b**) signals in chromosome 2 and 14, respectively. To the left is the P value related to GSD in a log10 scale. SNPs with the highest association to GSD are colored purple. The insert panel denotes the imputed quality score (r2) and the appropriate SNPs are colored in the plot.

The top-ten candidate variants were tested for replication in an independent admixed Chilean population composed of 1,643 individuals (626 cases and 1,017 controls) from Santiago de Chile (Table 1). Replication was performed by real-time qPCR with TaqMan probes for most leading variants, with the exception of SNP3 (4q12), SNP4 (*TRAF3*) and SNP9 (11p15.3), which were examined by proxy SNPs in high linkage disequilibrium (LD r^2^ > 0.88). Nine of the ten candidates were in Hardy-Weinberg equilibrium, where only the SNP3 showed a significant deviation (p = 2 × 10^−40^, Table 2), therefore association results from this candidate should be taken with caution. After genotyping and logistic regression analyses adjusted for age (in years; cases = 59.57 ± 12.47, controls = 48.26 ± 12.19, one-way ANOVA P < 0.001), sex (women%; cases = 84.82%, controls = 63.12%, Z score test P < 0.001), BMI (cases = 30.74 ± 5.92, controls = 28.61 ± 5.38, one-way ANOVA P < 0.001) and type 2 diabetes (T2D%; cases = 40.73%, controls = 14.36%, Z score test P < 0.001), we successfully replicated GSD association only of selected variants within the *ABCG8* (rs11887534, P = 0.001, OR = 1.59, CI 95% = 1.20-2.11) and *TRAF3* (rs12882491, P = 0.003, OR = 1.30, CI 95% = 1.09–1.54) genes (Fig. 2, Table 2). Both variants passed Bonferroni correction for 10 tests (P < 0.005) and displayed an effect with the same direction as it was observed in the discovery stage of the GWAS.

To assess for the combined effect between *ABCG8* and *TRAF3* SNPs with GSD, we performed a meta-analysis^17^ between the Discovery and Replication stages and observed an increased significance in the association of *ABCG8* (rs11887534, P = 3.24 × 10^−8^, OR = 1.74, CI 95% = 1.45–2.02) and *TRAF3* (rs12882491, P = 1.11 × 10^−7^, OR = 1.40, CI 95% = 1.20–1.60) variants (Table 2). Notably, the OR obtained for *ABCG8* represents a PAR of 7.1%, while for *TRAF3* (rs12882491, risk allele C) the OR of 1.40 (CI 95% = 1.20–1.60) represents a PAR of 20.2%. Taking together, the combined effect of both associations gives an estimated PAR of 25.9%, meaning that if their contribution for the disease is hypothetically eliminated^18^, there would be 25.9% fewer affected individuals in the population. In addition, SNPxSNP epistasis analysis showed non-significant interaction between *ABCG8* and *TRAF3* association signals (P = 0.399), suggesting that the genetic contribution of both risk factors is independent from each other. Altogether these results confirm and replicate the previously observed associations of *ABCG8* with GSD and identify *TRAF3* as a novel candidate gene associated with the disease in the Chilean Admixed population.

### *ABCG8* and *TRAF3* variants are associated with GBC

**Figure 3:**
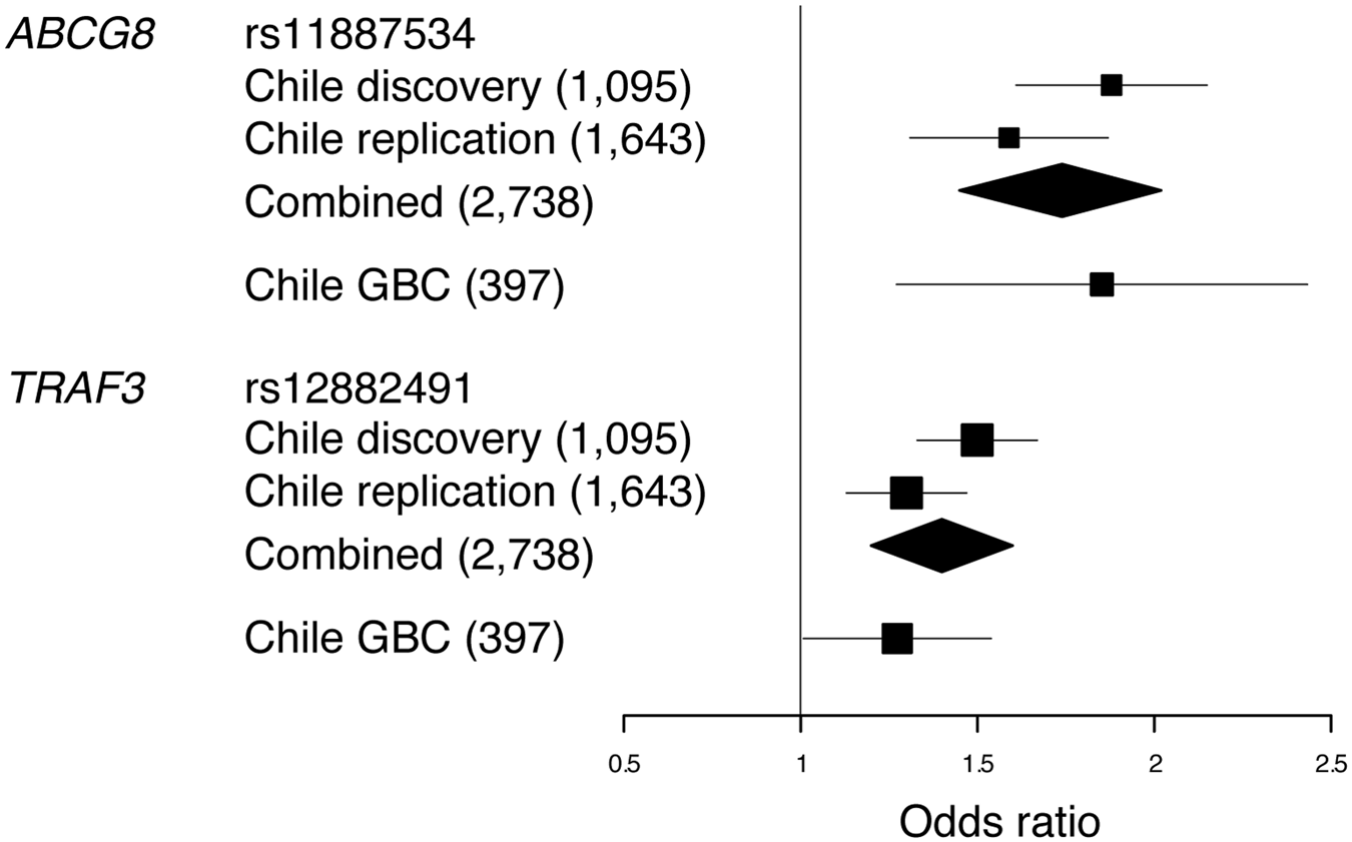
Effect size for *ABCG8* and *TRAF3* association signals in admixed Chilean populations. Forest plot calculated for GSD and GBC samples. The effect is shown in OR values and their 95% confidence intervals. Sample size for GSD case-control populations and for GBC cases are shown in parenthesis.

Although GBC is the most severe complication of GSD^5,6,19^, few studies have investigated the effect of *ABCG8* as a genetic determinant for this cancer^20,21^. We therefore examined the orientation and magnitude of the effect for *ABCG8* and *TRAF3* variants in a Chilean population of GBC patients (n = 397 women), using sex-matched controls from the replication population (n = 667 women) (Table 1). After logistic regression analyses adjusted by age (in years; cases = 62.65 ± 13.08, controls = 43.38 ± 12.13, one-way ANOVA P < 0.001), we observed that both SNPs were associated with GBC (*ABCG8* rs11887534: P = 6.9 × 10^−4^, OR = 1.77, CI 95% = 1.27–2.45; *TRAF3* rs12882491: P = 0.045, OR = 1.24, CI 95% = 1.004–1.53), with same direction of the effect and similar risk allele frequency (RAF), as it was observed in the discovery and replication populations (RAF_*ABCG8*_: GBC = 0.14, discovery = 0.14, replication = 0.12; RAF_*TRAF3*_: GBC = 0.66, discovery = 0.69, replication = 0.66) (Fig. 3). We also observed that the association of ABCG8 and TRAF3 variants with GBC was significant only when GBC samples were compared to gallstone-free individuals (Supplementary Table 2), indicating that the risk contribution to GBC was explained by the presence of gallstones. These results support the genetic association of *ABCG8* with GBC and reveal *TRAF3* as a novel marker for the disease.

### Replication Analyses for *ABCG8* and *TRAF3* variants in Europeans populations

Since we originally identified *ABCG8* as a risk factor for GSD in a German population from Kiel^10,11^, we therefore examined *ABCG8* rs11887534 and *TRAF3* rs12882491 variants in an extended sample from the original POPGEN-Kiel population consisting in 1,938 individuals (1,027 cases and 911 controls)^22^, as well as in 4,154 individuals (882 cases and 3,272 controls) from the north-east side of Germany identified as SHIP-Greifswald^23^ (Table 1). We found that *ABCG8* rs11887534 replicated both in POPGEN-Kiel (P = 5.36 × 10^−10^, OR = 2.00, CI 95% = 1.61–2.50) and SHIP-Greifswald (P = 1.29 × 10^−6^, OR = 1.87, CI 95% = 1.45–2.41) populations. Interestingly, no replication was observed for *TRAF3* rs12882491 either in the POPGEN-Kiel (P = 0.65, OR = 0.97, CI 95% = 0.84–1.11) or the SHIP-Greifswald (P = 0.39, OR = 1.06, CI 95% = 0.93–1.20) samples. Moreover, a difference in orientation and/or magnitude of the effect with the Chilean samples was also reflected in terms of risk allele frequency (RAF_*TRAF3*_ Chilean discovery = 0.69, replication = 0.66, POPGEN-Kiel = 0.34, SHIP-Greifswald = 0.35). Altogether, these results demonstrate a consistent effect of *ABCG8* association with GSD and indicate an ethnic-specific effect for the *TRAF3* variant in admixed Latinos with Native Amerindian ancestry.

### TRAF3 expression in human gallbladder and duodenal samples

**Figure 4:**
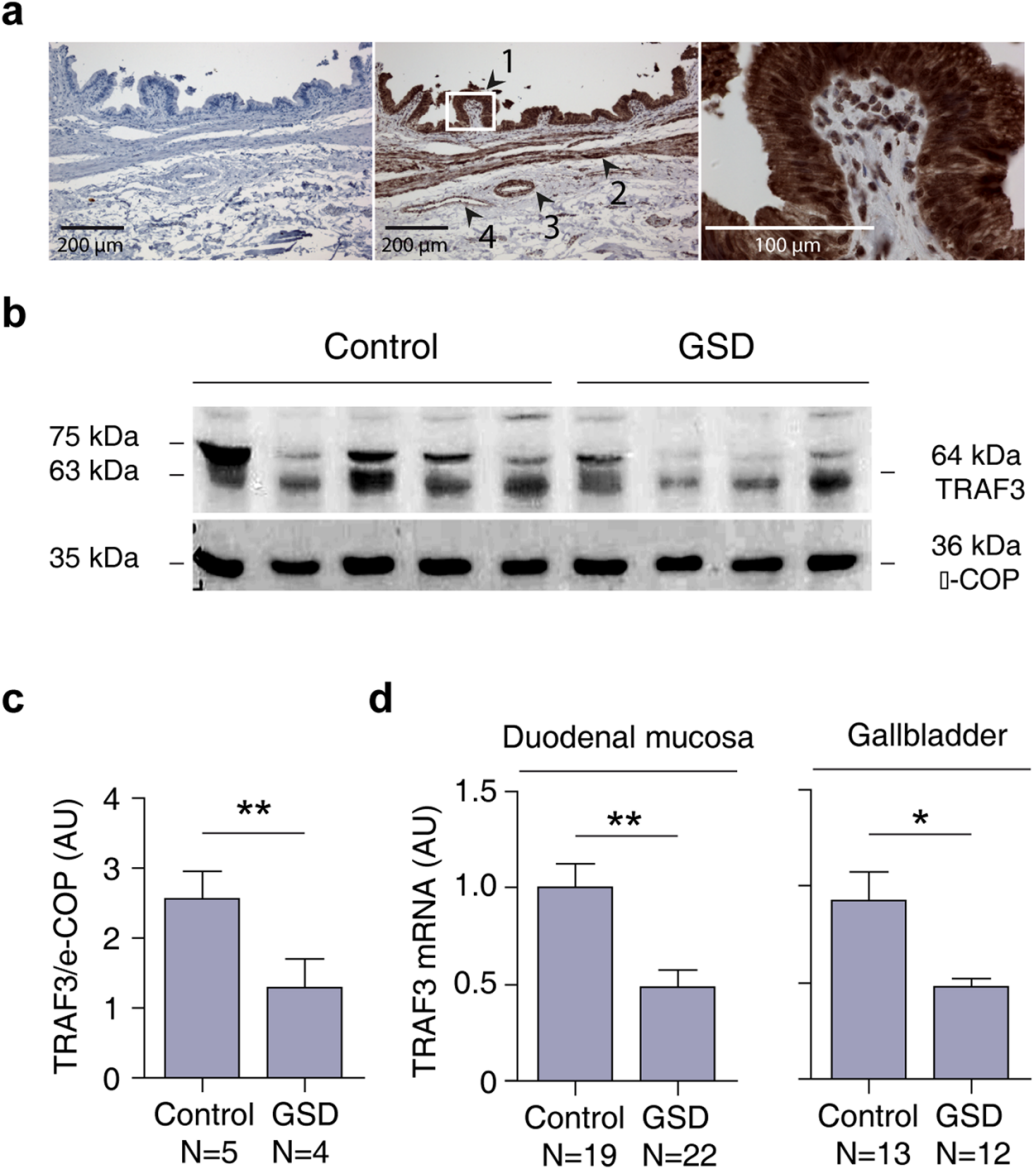
TRAF3 expression in human gallbladder and duodenal tissues. (**a**) Immunohistochemical analysis of TRAF3 expression in normal human gallbladder. Left panel: Negative control without primary antibody; Middle panel: Positive staining was observed for TRAF3 in the cell epithelium (1), muscle fibers (2), arterioles (3), and veins (4); Right panel: Zoom-in (60x) showing TRAF3 localization in the mucosal epithelium. (**b**) Western blot for TRAF3 in biopsies of duodenal mucosa tissue from GSD cases and control individuals. (**c**) Determination of TRAF3 protein levels in the duodenal mucosa (as is observed in b). (**d**) Differential expression analyses for TRAF3 mRNA in duodenal (left panel) and gallbladder mucosa tissues (right panel) in GSD cases and control samples.

*TRAF3* is a member of the tumor necrosis factor (TNF) receptor-associated factor (TRAF) protein family that mediates signal transduction during the activation of immune and inflammatory responses^24–26^. To date, seven *TRAF* genes are known in humans (*TRAF1* to *TRAF7*) and the products encoded by these genes are involved in cytokine production and cell survival^27^. Considering that the acute and chronic inflammation of the gallbladder (cholecystitis) are known hallmarks of GSD pathophysiology^28^, we explored the expression levels of the *TRAF3* protein in 9 gallbladder mucosa samples (4 cases and 5 controls) and mRNA in 25 gallbladder (12 cases and 13 controls) and 41 duodenal (22 cases and 19 controls, independent from the mRNA samples) samples. We used the duodenal mucosa as a comparison for three reasons: First, it is a tissue in contact with bile coming from the liver and gallbladder; second, it is easily accessible by endoscopy in order to obtain biopsies from affected and healthy individuals; and third, evidence show the existence of a similar gene expression pattern compared with both liver and gallbladder tissues^29^. Immunohistochemical staining in the gallbladder mucosa revealed that TRAF3 protein was observed in different gallbladder cell types such as the epithelium, smooth muscle fibers, arterioles and veins (Fig. 4a). Notably, we found a significant decrease of protein levels in the duodenal mucosa of GSD cases compared to control individuals (P < 0.001, two-tailed t-test; Fig. 4b,c and Supplementary Fig. 3) and also observed a significant decrease of the gene transcripts in the set of mRNA samples from duodenal (P < 0.001, two-tailed t-test) and gallbladder mucosa (P = 0.015, two-tailed t-test) (Fig. 4d). These results show lower levels of TRAF3 in GSD affected individuals, suggesting a functional contribution of this gene during the inflammatory response observed during the onset or development of the disease.

## Discussion

Herein we report that common genetic variants within the *ABCG8* and *TRAF3* genes confer risk to GSD and GBC in the admixed Chilean population. The *ABCG8* polymorphism rs11887534 (D19H) has been reported in several world populations including Latinos^15^, thus the results reported by the present study serves as a novel replication and adds strength to the observed association of the gene. In the discovery stage, the leading *ABCG8* SNP showed a lower magnitude of association, compared with the estimated from the German population^10^, which could be explained by the difference in the risk allele frequency (C allele) between Chilean and German controls (0.079 vs. 0.05, respectively). This could be a reason for not being able to detect in the discovery stage a genome-wide signal (P < 5 × 10^−8^) in the *ABCG8* locus, suggesting that larger sample sizes are required to reach such significance level in future studies on this population.

*ABCG8* was consistently associated with GSD in all the populations analyzed in this study, however, *TRAF3* association was only observed in Chilean samples. These results suggested that TRAF3 association might have an ethnic-specific effect. Interestingly, data from the 1000 genomes project from Peru (PEL), Mexico (MXL), Colombia (CLM) and Puerto Rico (PUR) show a higher risk allele frequency compared with the one from the overall European population (RAF PEL = 0.84, MXL = 0.55, CLM = 0.53, PUR = 0.49, EUR = 0.34) indicating that would be of interest to validate the *TRAF3* association in closely related Latino populations like Peru, Argentina and Bolivia, that also have a high GSD prevalence^30–32^.

GSD has known confounding factors such as age, gender, BMI and T2D, and past studies have considered some of these biological variables into their association analyses^10,15,33,34^. Furthermore, admixed populations present a special challenge in GWAS analyses due to the presence of 2 or more ancestry components that needs to be considered when performing the association testing^35,36^. In this regard, we performed 2 additional analyses to examine whether the association of *ABCG8* and *TRAF3* variants with GSD could be affected by these confounding factors. First, besides age as the main covariate, we included the 5 first eigenvectors from the principal component analysis (PCA, see methods) to take into account any hidden population structure that could still be present. Second, we adjusted the logistic regression by age, the aforementioned 5 eigenvectors and the other known GSD covariates such as gender and BMI to test their effect regardless of their distribution in cases and controls. Remarkably, we found that these additional adjustments did not alter the selection of *ABCG8* or *TRAF3* as candidate genes for the replication stage since the association P values for their leading variants remained above the significance cutoff of P < 1 × 10^−5^ (Supplementary Table 3).

Here we also observed that *ABCG8* and *TRAF3* variants are significantly associated with GBC in admixed Native Americans. For instance, the *ABCG8* rs11887534 variant has been previously associated with this malignancy in China^21^, India^20^ and European populations^37^ and most of the studies show that the GBC risk is more pronounced in patients that also had biliary stones in the gallbladder, as it is likely observed in this Chilean cohort (i.e. composed of 90% women GBC patients with cholesterol gallstones). This agrees with our findings where both *ABCG8* and *TRAF3* variants only contribute to risk when comparing GBC subjects with gallstone free controls^38^. A recent report from a group in India showed a GWAS scan for GBC in their population and found that the genes *ABCB1* and *ABCB4* were significantly associated with GBC^38^. We looked for their association in our GSD GWAS discovery population and found that none of the 3 reported SNPs showed at least nominally significant P values (Supplementary Table 4). This could be indicating that these new GBC risk factors are not be necessarily associated to the GSD phenotype linked to GBC in the Chilean population.

TRAF3 belongs to the TNF receptor associated factor (TRAF) protein. TRAF3 function has been related to the promotion of autoimmunity and predisposition to various cancers^39^, including multiple myeloma^40^, Hodgkin lymphoma^41^ and splenic neoplasms^42^. The protein physically interacts with the NF-κB-inducing kinase (NIK) promoting NIK degradation by the proteasome, which results in the inactivation of the non-canonical NF-κB pathway^43^. Indeed, siRNA knockdown of *TRAF3* in human cancer cell lines stabilizes NIK and activates NF-κB dependent transcription^44^. Interestingly, recent evidence indicates that activation of NF-κB results in invasion, lymphangiogenesis and tumor growth in GBC tissue and cell lines^45,46^, and therefore genetic alterations in one of its modulators could be directly involved in GBC pathogenesis and may serve as a target of future therapies and prevention.

Inflammation is a hallmark of GSD pathogenesis and it has been suggested that the formation of biliary stones is preceded by histopathologic alterations in the gallbladder wall that indicate tissue inflammation^47^, including edema, increased wall thickness, decreased motility and altered transport function in the epithelium. Therefore, a crosstalk between cholesterol stone formation and inflammatory processes might exist that promote the development or progression of the disease and suggests that *ABCG8* and *TRAF3* could be both relevant actors in the pathology. First, it is known that the *ABCG8* variant promotes gallstone formation by enhancing the efflux of cholesterol-saturated bile^11^. Second, lower expression levels of TRAF3 in duodenal mucosa and gallbladder epithelium of GSD patients (as observed in the present study) could be responsible for increased production of pro-inflammatory factors, such as NF-κB, IL-6 and IL-12B, as well as for decreased activity of anti-inflammatory factors such as type 1 interferon and IL-10^44,48–50^. Notably, the IL-10 has already been associated with GSD and GBC^51^. Interestingly, according to the Genotype-Tissue Expression (GTEx) project database, which offers gene expression and quantitative trait loci associations from 53 human tissues^52^, individuals with the *TRAF3* risk allele (rs12882491, C) present decreased TRAF3 expression levels in the small intestine, the closer tissue to our duodenum data (Supplementary Fig. 4). Although the correlation is not statistically significant, the direction of the effect agrees with our findings, indicating that the GWAS risk variant could be functionally linked to the decreased gene levels observed in the study.

In summary, our results confirm the association of *ABCG8* and identify *TRAF3* as a novel candidate gene for GSD and GBC in the high-risk Chilean admixed population. We hypothesize that both genes are functionally involved in the cascade of events that triggers gallstone formation and the inflammatory response observed in affected patients. Further research is needed to determine the role of the variations within the *TRAF3* gene that could explain the observed differential gene expression and its contribution to the pathophysiology of the disease.

## Methods

### Ethical statement

All procedures involving genetic and clinical data usage from Chilean GSD and GBC patients were approved by the Ethical Scientific Committee at the Pontificia Universidad Catolica de Chile and were conducted in accordance with the guidelines of the National Commission on Science and Technology (CONICYT-Chile). All participants provided informed consent. In the German replication samples, Ethical Scientific Committees have previously approved these studies^22,53^.

### Study participants

#### Discovery stage

The Chilean GWAS discovery population consisted in 1,235 individuals, belonging to the ANCORA family health centers in Santiago de Chile (Puente Alto, La Florida and La Pintana). This sample constitutes an admixed group (European-Amerindian) aged between 20–80 years old, of middle-low income, from an urban area and representative of the general Chilean population. Cases were defined as patients with reported cholecystectomy or by the presence of gallstones in their gallbladder assessed by ultrasound. Control individuals had normal gallbladders, with neither a history of gallbladder surgery nor gallstone findings in ultrasound. Since women have a higher risk to develop gallstones compared to men, the population was mainly composed of women (1,013 individuals: 489 cases and 524 controls), corresponding to the 93.05% of the sample. After genotyping and QCs (see ahead), 1,095 individuals remained for further analysis (529 cases and 566 controls, Table 1). To reduce the contribution of confounding factor in the following genetic association analyses, cases and controls were matched for known GSD covariates and comorbidities, including age at sampling, sex distribution and BMI (Table 1). We excluded T2D individuals from the discovery stage in order to reduce the number of factors to consider in the adjusted analyses.

#### Chilean GSD replication stage

The independent replication population consisted of 1,643 Chilean individuals (626 cases and 1,017 controls), coming from the ANCORA family health centers and from a population base study in La Florida, which has been previously described^7^. Information regarding age, sex, BMI and T2D was gathered for all individuals to control for covariates/comorbidities in the genetic association analyses.

#### Chilean gallbladder cancer (GBC) population

A secondary Chilean replication population was derived from GBC patients, with more than 90% presenting gallstones. The population is composed of a total number of 397 incident and prevalent women cases, recruited from different health centers of the country including: Hospital Sótero del Río, Clinical Hospital of the Pontificia Universidad Católica de Chile, Hospital Regional de Valdivia, Hospital Regional de Temuco and Hospital Regional de Concepción. GBC phenotype was defined according to criteria and steps described by the Classification of Malignant Tumors and the American Joint Committee on Cancer (TNM-AJCC) where only adenocarcinomas were included. Each histological cut was reviewed and selected by two independent expert pathologists. After delimiting the healthy tissue, either adjacent to the tumor or other non-tumoral tissues such as liver or cystic ganglion, blocks were selected for sequential cuts of 10 µm thickness and used for extraction of genomic DNA. DNA concentration and integrity were determined by the NanoDrop ND-1000® Spectrophotometer and by agarose gel electrophoresis, respectively.

#### European replication populations

We analyzed two European GSD population from Germany. The first population comes from the POPGEN biobank of the Medicine Faculty at the University of Kiel, consisting in 1,938 individuals (1,027 cases and 911 healthy controls) where GSD status was determined by cholecystectomy or ultrasound^22^. Considering that GSD prevalence increases with age^10^, the statistical analysis performed on this sample did not include age as a covariate, since stone-free controls were chosen and they had a higher median age (59.43 years old) than affected individuals (45.5 years old). The second population comes from the Study of Health in Pomerania (SHIP) from the Greifswald region in northeast Germany, comprising 4,154 subjects (882 GSD cases and 3,272 healthy controls) where GSD cases were identified by cholecystectomy/ultrasound and control individuals by senlf-reports^23^.

### Genomic DNA extraction and genotyping

Genomic DNA was extracted from samples of the Chilean discovery population, replication and GBC incident cases (alive patients) using peripheral blood (Invisorb Blood Universal kit, *Invitek*, Berlin, Germany). For GBC prevalent cases, DNA was extracted from normal (non-tumoral) tissue samples using the QIAamp DNA-Mini-Kit © (Qiagen, Mainz, Germany). Extracted DNA was quantified with a nanodrop spectrophotometer to a fixed concentration of 50 ng/μl. For the 2 European populations, DNA extraction methods is described in their respective publications. Genome-wide genotyping on the Chilean discovery population was performed with the Affymetrix Axiom® Genome-Wide LAT 1 World Array 4 at the Cologne Center for Genomics (University of Cologne, Germany). The microarray includes probes to identify 818,155 SNPs and it has been optimized to enhance the capture of variants in populations with Amerindian ancestry^54^. Genotype calling was done with Affymetrix Power Tools software, including the SNPolisher v.1.17.0 R package. For the POPGEN-Kiel and SHIP-Greifswald European populations, the Axiom® Genome-Wide Platform EUR 1 = (674,518 markers) and the Affymetrix Genome-Wide Human SNP Array 6.0 (1,879,489 markers) were used for genome-wide genotyping, respectively. For the Chilean replication and GBC populations, genotyping was carried out by real-time quantitative PCR (qPCR) using TaqMan probes and the TaqMan Universal Master Mix (Thermo Fisher Scientific Inc, Foster City, USA). qPCR reactions and genotype calling were performed in the StepOnePlus^TM^ System (Thermo Fisher Scientific Inc, Foster City, USA).

### GWAS quality control procedures

After genotyping of the initial 1,235 samples for the Chilean discovery stage, we used the Affymetrix Power tools protocol and removed 6 individuals and 5,136 variants, leaving 1,229 subjects and 813,019 genotypes, then converted them to PLINK v1.9 format^55,56^ for further quality controls^57^. We performed an identity by descent (IBD) analysis to detect cryptic relationships in the GWAS, identifying 115 individuals having an IBD score > 0.185, indicating close relatedness (2nd degree and further). In order to detect population substructure, we performed a principal component analysis (PCA) with the SMARTPCA tool included in the EIGENSOFT 6.0.1 package software^58^ using the 1000 genome project phase 3 as a reference population, similar to what was described in a recent study that included the present GWAS cohort^59^. We identified 15 individuals as outliers (>6 standard deviations) and then used the first 5 eigenvectors as covariates in an extended GWAS analysis. One individual had inconsistent sex identification (PLINK sex check), eight had high genotype heterozygosity rates (>5 standard deviations) and no individuals had < 97% of genotype successfully called. Combining all the individuals detected with the methods described above, we obtained a total number of 134 unique samples and excluded them, leaving 1,095 good quality samples (529 cases and 566 controls). At the variant level, we identified 2,940 SNPs deviating from Hardy-Weinberg equilibrium (HWE < 1 × 10^-6^) in controls, 170 SNPs showing 1% or more genotype missingness (GENO > 0.01) and 122,074 SNPs had a minor allele frequency of less than a 1% in the total population (MAF < 0.01), giving a total number of 125,184 SNPs removed, and leaving 677,835 for further analysis. For the POPGEN-Kiel population, samples were imputed with IMPUTE2 using the 1000 Genomes phase 3 reference panel (build 37). TRAF3 and ABCG8 variants were selected from all variants surpassing MAF > 0.01 and an INFO > 0.4. For the SHIP-Greifswald population, TRAF3 and ABCG8 variants summary statistics were obtained from the study authors^53^.

### Global Ancestry Estimation

Global ancestry proportions for each individual in the Chilean discovery population was calculated with the ADMIXTURE v.1.3.0 software^60^ using the reference panel of the 1000 Genomes project Phase-3 and 11 individuals from the Mapuche-Huilliche ethnic group as a Chilean Native American reference panel (HUI)^59^. The mean proportion of the Amerindian ancestry was calculated in cases and controls and tested for significant deviation using a one-way ANOVA test.

### Genotype imputation and GWAS

Genotypes were imputed using IMPUTE2 v2.3.1 without the prephasing step in order to gain accuracy^61,62^. We used the genotype reference panel coming from the 1000 Genomes Project Phase 3 for autosomic variation (October 2014 release) and 1000 Genomes Project Phase 1 for the X chromosome (March 2012 release). After imputation, we selected SNPs and Indels with an INFO score > 0.4, MAF > 0.01 and HWE > 1 × 10^−6^ leaving 9,433,911 analysis-ready variants. Information on whether a candidate variant comes from imputation or actual chip genotyping is presented in Table 1. Genetic association analyses were performed using the score statistic as an additive logistic regression model implemented in SNPTEST v2.5^63^ and adjusted for age as a covariate. Other GSD known confounding factors such as gender, BMI and TD2 were either matched or non-existent in cases and controls (see results), therefore we did not include them in the main association analysis in order to avoid overfitting. The Population Attributable Risk (PAR) was calculated for *ABCG8* and *TRAF3* leading SNPs in the combined Chilean discovery and replication population, using the formula: PAR = P_RF_ × (RR − 1)/1 + P_RF_ × (RR − 1) × 100, where P_RF_ is the prevalence of the risk factor in the general population. The allelic odds ratio (OR) was used as an approximation for the estimated relative risk (RR) of disease due to exposure to the risk allele. Combined PAR for the 2 risk variants was calculated as: Combined PAR = 1 − (1 − PAR_1_) ×(1 − PAR_2_). SNPxSNP interaction analysis was performed with PLINK v1.9 epistasis function.

### Genotyping in replication analyses

TaqMan probes used for the 10 candidate variants coming from the discovery stage are shown in the Supplementary Table 5. The *TRAF3* leading indel (rs368550004) was genotyped using a high resolution melting (HRM) procedure, as described elsewhere^64^. Briefly, HRM was performed using specific DNA primers for TRAF3 (Forward primer = 5′-TCACATTCCATACAATTACC-3′, reverse primer = 5′-ATTAACAAGGAACAACCGAT-3′) and MeltDoctor High-Resolution Melting (HRM) Reagents (Applied Biosystems, cat: 4415440).

### Statistical analyses in the replication populations

Replication association analysis was performed using additive logistic regression tests with PLINK v1.9, adjusting by age, sex, BMI and T2D status as they were available for the data. Fixed effect meta-analysis was performed between the discovery stage and variants showing successful replication after Bonferroni correction for 10 tests (P < 0.005), using the inverse-variance method implemented in the *–meta-analysis* command in PLINK. Forest plots were generated using the rmeta package v.2.16 in R. Regional association plots were generated using LocusZoom (v1.1)^65^.

### Differential gene expression analysis of TRAF3

For expression and histological analyses (see below) human gallbladder mucosa samples were used as described^66^. Briefly, duodenal mucosa samples were extracted from gallstone and gallstone-free individuals that met the following criteria: women 18 to 35 years of age, non-obese (BMI 18–30 kg/m^2^), without significant pathologies and consumption of any drug 2 months prior to recruitment and not pregnant at the time of contact. Duodenal biopsies were taken from the second segment of the duodenum (distal to the ampulla of Vater), during gastrointestinal endoscopy performed at the Endoscopic Unit of the Gastroenterology Department of the Pontificia Univesidad Católica de Chile. Total RNA from was extracted using the PureLink^TM^ RNA Mini Kit (Ambion Life technologies, Carlsbad, USA) and reverse-transcribed into cDNA with a High-Capacity cDNA Reverse Transcription Kit (Thermo Fisher Scientific Inc., Carlsbad, USA). Quantitative RT-PCR from 25 gallbladders (12 cases and 13 controls) and 41 duodenal (22 cases and 19 controls) samples was performed using specific DNA primers for TRAF3 (Forward primer = 5′- TACAGCGTGTCAAGAGAGCATCGT-3′, reverse primer = 5′- CACAACCTCTGCTTTCATTCCGACAATAG-3′) and SYBR-Green Brilliant III Ultra-Fast SYBR-Green qPCR Master Mix (Agilent Technologies Inc., Santa Clara, USA). Data were expressed in arbitrary units and were normalized to *RNA18S* levels. Statistical significance was determined using a two-tailed t-test (*P < 0.05).

### Western blot

Fifty micrograms of total proteins from duodenal mucosa lysate of 9 samples (5 control and 4 cases, all independent from the mRNA samples), was separated on a 12% SDS-PAGE gel and then transferred onto nitrocellulose membranes (Millipore, Billerica, MA). Using 5% nonfat dry milk for blocking, membranes were incubated overnight at 4 °C with rabbit anti-TRAF3 antibody (1:1000, ABCAM Ltd, Cambridge, UK) and with rabbit polyclonal *ε-*Cop antibody (1:5000, donated by Dr. Monty Krieger of the Biology Department, Massachusetts Institute of Technology, Cambridge, USA) for 1 h at room temperature. Goat anti-rabbit IgG-HRP were used as secondary antibodies (1:1000, Santa Cruz Biotechnology, Inc., Santa Cruz, CA, USA) for 1 h at room temperature. The signals were detected using the chemiluminescence kit ECL Western Blotting (Pierce Biotechnology, Inc., Rockford, IL, USA). Complete Western Blot gels from where Fig. 4b. was composed is presented in the Supplementary Fig. 3.

### Immunohistochemistry analysis

Two-micron sections of gallbladder samples were fixed with formalin and embedded in paraffin. For TRAF3 immunohistochemistry analysis, the rabbit anti-TRAF3-ab155298 antibody was used (1:100, ABCAM, Ltd, Cambridge, UK) and was visualized using the Envision Flex/HRP SM802 complex method (Dako, Agilent Technologies Company, Santa Clara, CA 95051, USA). Labeled sections were examined and captured using an Olympus BX51 microscope (Olympus, Tokyo, Japan) with the software Stereo Investigator v.11.03 (MBF bioscience, Williston, VT, USA) and analyzed with the Image-Pro Express program (Media Cybernetics, Bethesda, MD, USA) as described previously^66^.

## Electronic supplementary material

Supplementary Information

## Data Availability

All genotype files for the Chilean discovery population are available upon request to the corresponding authors.
