# Supplementary material for: Variants in ABCG8 and TRAF3 genes confer risk for gallstone disease in admixed Latinos with Mapuche Native American ancestry
Source: Sci Rep. 2019 Jan 28;9:772. doi: 10.1038/s41598-018-35852-z (PMC6349870; doi:10.1038/s41598-018-35852-z)
Supplement: Supplementary file 1 — Supplementary Information [file 41598_2018_35852_MOESM1_ESM.docx]

**Supplementary Information**

**Variants in *ABCG8* and *TRAF3* genes confer risk for gallstone disease in admixed Latinos with Mapuche Native American ancestry**

Bernabé I. Bustos, Eduardo Pérez-Palma, Stephan Buch, Lorena Azócar, Eleodoro Riveras, Giorgia D. Ugarte, Mohammad Toliat, Peter Nürnberg, Wolfgang Lieb, Andre Franke, Sebastian Hinz, Greta Burmeister, Witigo von Schönfels, Clemens Schafmayer, Henry Völzke, Uwe Völker, Georg Homuth, Markus M. Lerch, José Luis Santos, Klaus Puschel, Claudia Bambs, Juan Carlos Roa, Rodrigo A. Gutiérrez, Jochen Hampe, Giancarlo V. De Ferrari^*^ and Juan Francisco Miquel^*^.

*Corresponding authors: Drs. Juan Francisco Miquel ([jfmiquel@med.puc.cl](mailto:jfmiquel@med.puc.cl)) and Giancarlo V. De Ferrari ([gdeferrari@unab.cl](mailto:gdeferrari@unab.cl)).

**Supplementary Table 1.** Chilean GWAS results for known GSD risk factors discovered in Joshi et al. (2016) meta-analysis.

|  | | | Joshi et al. 2016 GSD meta-analysis results | | | | Chilean GWAS Results | | | |
| --- | --- | --- | --- | --- | --- | --- | --- | --- | --- | --- |
| CHR | Gene | SNP | RA | RAF | *P* value | OR (95% CI) | RA | RAF | *P* value | OR (95% CI) |
| 2 | GCKR (P446L) | rs1260326 | C | 0.61 | 7.74x10^-8^ | 1.12 (1.09-1.15) | C | 0.31 | 0.07 | 1.18 (0.99-1.41) |
| 2 | ABCG8 (D19H) | rs11887534 | C | 0.07 | 1.99x10^-75^ | 1.78 (1.70-1.86) | C | 0.14 | 5.24x10^-6^ | 1.88 (1.43-2.47) |
| 2 | ABCG8 (intron) | rs4245791 | T | 0.70 | 5.29x10^-31^ | 1.28 (1.25-1.32) | T | 0.15 | 5.49x10^-5^ | 1.56 (1.26-1.94) |
| 3 | TM4SF4 (intron) | rs9843304 | C | 0.45 | 3.00x10^-6^ | 1.11 (1.08-1.14) | T | 0.37 | 0.96 | 1.00 (0.85-1.19) |
| 8 | CYP7A1/UBXN2B (intergenic) | rs6471717 | G | 0.34 | 3.16x10^-6^ | 1.11 (1.08-1.14) | G | 0.23 | 0.23 | 1.13 (0.92-1.39) |
| 19 | SULT2A1 (intron) | rs2547231 | A | 0.84 | 1.09x10^-7^ | 1.17 (1.13-1.22) | A | 0.07 | 0.02 | 1.43 (1.06-1.94) |

CHR = Chromosome; SNP = Single Nucleotide Polymorphism; BP = Base pair position; RA = Risk allele; RAF = Risk allele Frequency in cases

**Supplementary Table 2.** Association analysis of ABCG8 and TRAF3 risk SNPs in GBC cases (N=390) vs GSD individuals (N=560).

| SNP:GENE | RA | RAF GBC | RAF GSD | P | OR |
| --- | --- | --- | --- | --- | --- |
| rs11887534:ABCG8 | C | 0.140 | 0.126 | 0.286 | 1.16 |
| rs12882491:TRAF3 | C | 0.656 | 0.648 | 0.589 | 1.05 |

SNP = Single Nucleotide Polymorphism; RA = Risk allele; RAF = Risk allele Frequency

**Supplementary Table 3.** Logistic regression P values for the discovery candidates adjusting by different GSD covariates.

| Candidate | LOCUS | SNPID | HWE^a^ | AGE | AGE, PCA | AGE, GENDER, BMI, PCA |
| --- | --- | --- | --- | --- | --- | --- |
| 1 | ELMO1 | rs4446645 | 0.444 | 3.36x10-7 | 1.47E-06 | 1.03E-06 |
| 2 | 5q34 | rs10463138 | 0.438 | 2.47x10-6 | 1.06E-06 | 1.13E-06 |
| 3 | 4q12 | rs74537816 | 0.459 | 3.54x10-6 | 2.91E-06 | 1.21E-06 |
|  | 4q12 | rs1824387 | 0.419 | 3.95x10-5 | 2.68E-05 | 1.47E-05 |
| 4 | TRAF3 | rs368550004 | 0.335 | 3.92x10-6 | 8.43E-06 | 9.67E-06 |
|  | TRAF3 | rs12882491 | 0.383 | 5.28x10-6 | 1.04E-05 | 1.18E-05 |
| 5 | 9p21.1 | rs4879592 | 0.626 | 4.66x10-6 | 3.08E-06 | 3.40E-06 |
| 6 | OLFML2B | rs10918361 | 0.404 | 4.91x10-6 | 4.70E-06 | 2.25E-06 |
| 7 | 3p22.2 | rs73827633 | 0.640 | 5.06x10-6 | 5.59E-06 | 3.31E-06 |
| 8 | ABCG8 | rs11887534 | 1 | 5.24x10-6 | 4.63E-06 | 5.35E-06 |
| 9 | 11p15.3 | rs147367002 | 1 | 7.63x10-6 | 2.02E-05 | 2.79E-05 |
|  | 11p15.3 | rs16908929 | 0.168 | 4.30x10-5 | 9.66E-05 | 0.0001 |
| 10 | TRPV1 | rs7223530 | 0.328 | 8.07x10-6 | 1.43E-05 | 1.98E-05 |

**^a^Hardy-Weinberg equilibrium p-values calculated in control samples (discovery stage)**

**Supplementary Table 4.** Association analysis of GBC risk SNPs reported by Mahtre et al 2017 in the GSD GWAS discovery cohort.

| Gene | RA | SNP | RAF cases | RAF controls | P value | OR |
| --- | --- | --- | --- | --- | --- | --- |
| ABCB1 | A | rs17209837 | 0.85 | 0.83 | 0.205 | 1.16 |
| ABCB4 | A | rs1558375 | 0.81 | 0.80 | 0.541 | 1.01 |
| ABCB4 | A | rs4148808 | 0.84 | 0.83 | 0.403 | 1.10 |

SNP = Single Nucleotide Polymorphism; RA = Risk allele; RAF = Risk allele Frequency

**Supplementary Table 5.** TaqMan probes used in the GWAS replication cohort

| GWAS candidate | Locus | Rs ID | TaqMan ID |
| --- | --- | --- | --- |
| SNP 1 | *ELMO1* | rs4446645 | C_2869174_20 |
| SNP 2 | 5q34 | rs10463138 | C_1996438_10 |
| SNP 3 | 4q12 | rs1824387 | C_27316752_20 |
| SNP 4 | *TRAF3* | rs12882491 | C_11467183_10 |
| SNP 5 | 9p21.1 | rs4879592 | C_27921348_10 |
| SNP 6 | *OLFML2B* | rs10918361 | C_31086974_10 |
| SNP 7 | 3p22.2 | rs73827633 | C_98747530_10 |
| SNP 8 | *ABCG8* | rs11887534 | C_26135643_10 |
| SNP 9 | 11p15.3 | rs16908929 | C_33645573_10 |
| SNP 10 | *TRPV1* | rs7223530 | C_30054692_10 |


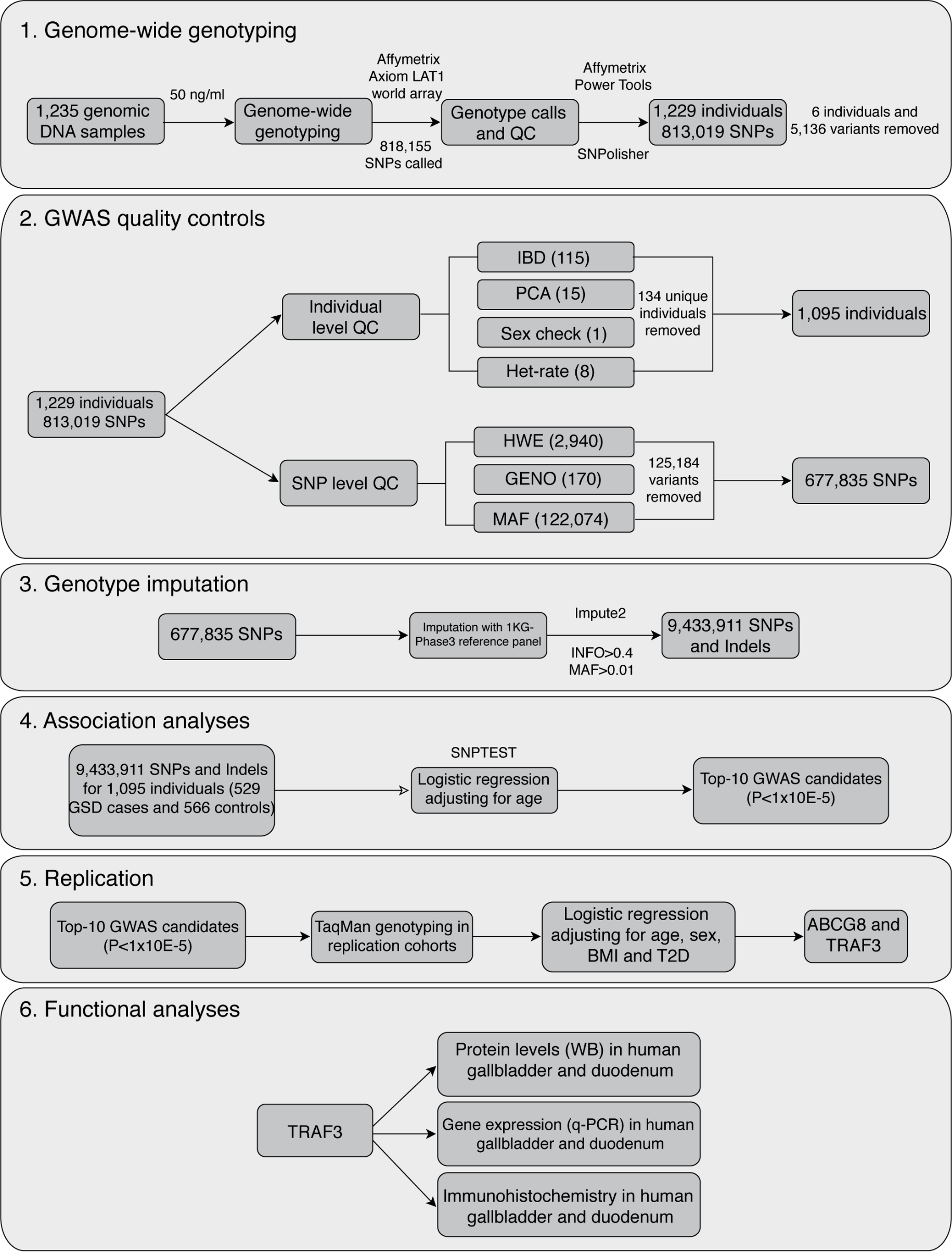


**Supplementary Figure 1. Experimental pipeline**. Main procedures performed in the study are depicted in the flowchart as follows: Genome-wide genotyping, variant calling and quality controls for the stage 1; further GWAS quality controls to reach the final number of individuals and variants for the analysis; genome-wide imputation of variants from 1000 genomes phase 3 reference panels; association analyses for stage 1; replication of the 10 top candidate hits and functional analyses for the *TRAF3* gene.


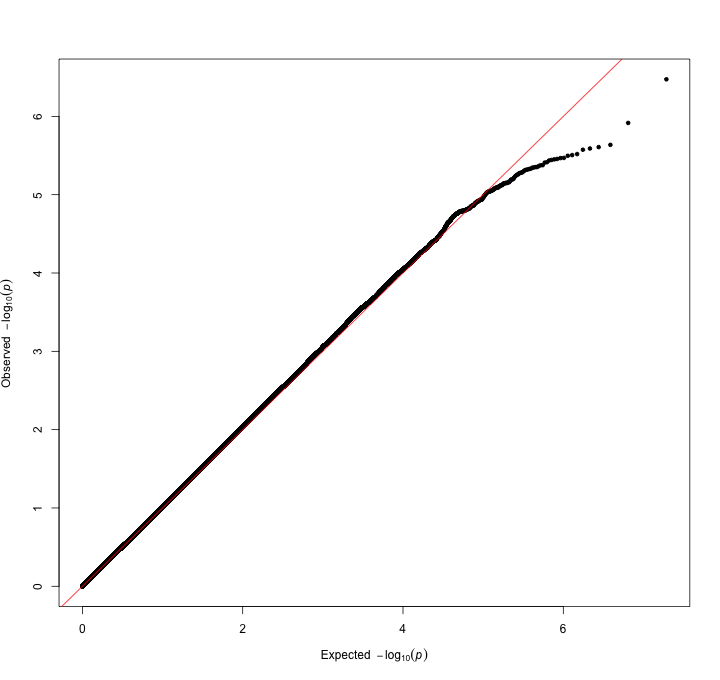


**Supplementary Figure 2. Quantile-Quantile plot for the genome-wide association results in the Discovery population.** The red diagonal line represents the expected values of association under the distribution of the GWAS *P* values (black dots), where at the end of the curve a deviation is observed due to the most significant results obtained which are represented as the inflation factor in the analysis (λ=1.02).


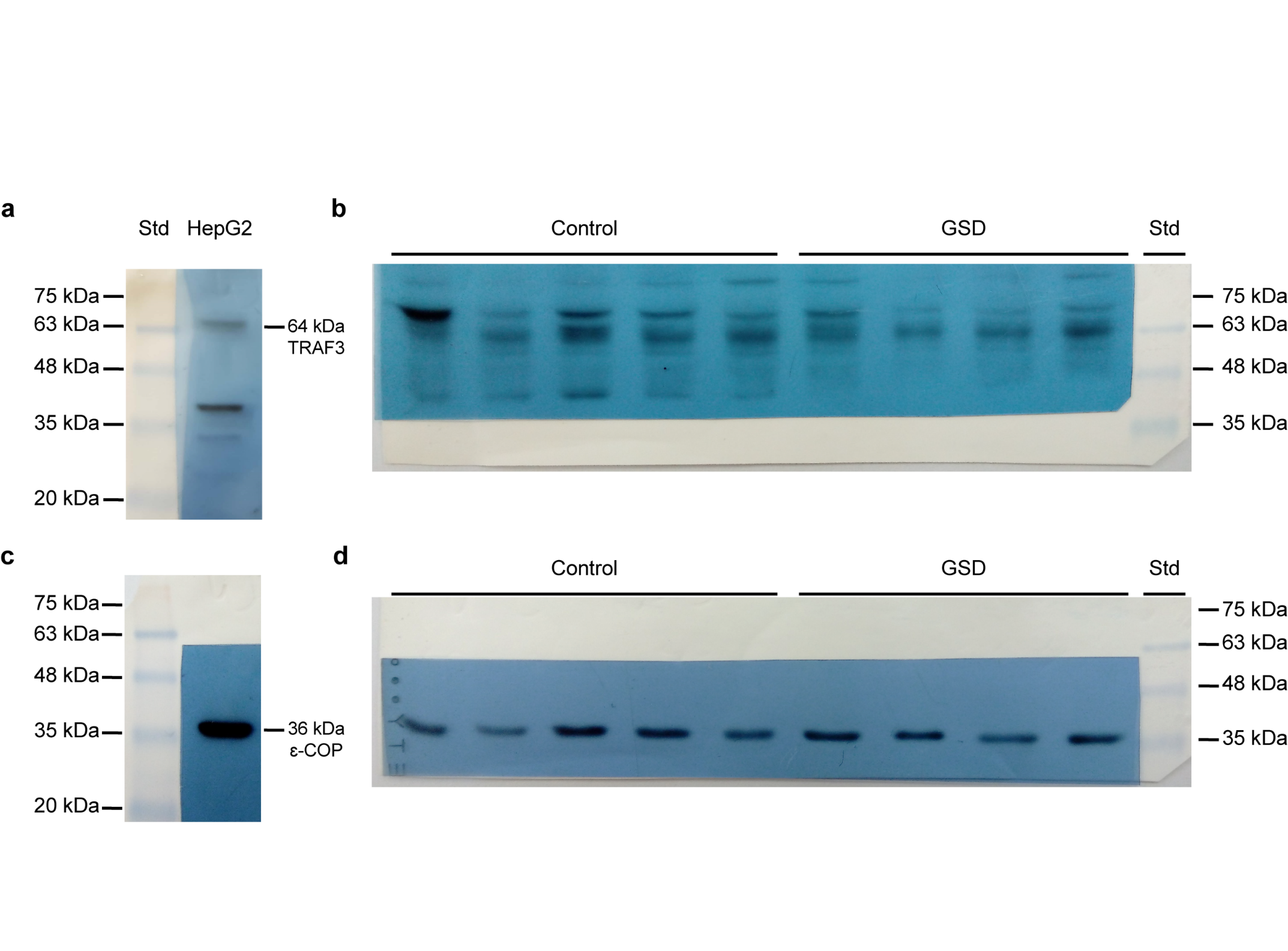


**Supplementary Figure 3.** **Western blot of TRAF3 protein in lysates of human duodenal tissues and HepG2 cells**. Fifty micrograms of total protein from human duodenal mucosa and twenty micrograms of total protein from HepG2 cells (positive control) were visualized by Western blot. (a) Positive control of TRAF3 (64 KDa) performed in HepG2 cells lysate**.** (b) TRAF3 protein levels were evaluated in 4 GSD and 5 healthy controls of human duodenal samples. (c) Positive control of ε-COP protein (36 kDa) in HepG2 cells lysate. (d) ε-COP protein levels evaluated in the GSD case/control human duodenal samples. All images were aligned between nitrocellulose membranes and their films.

**
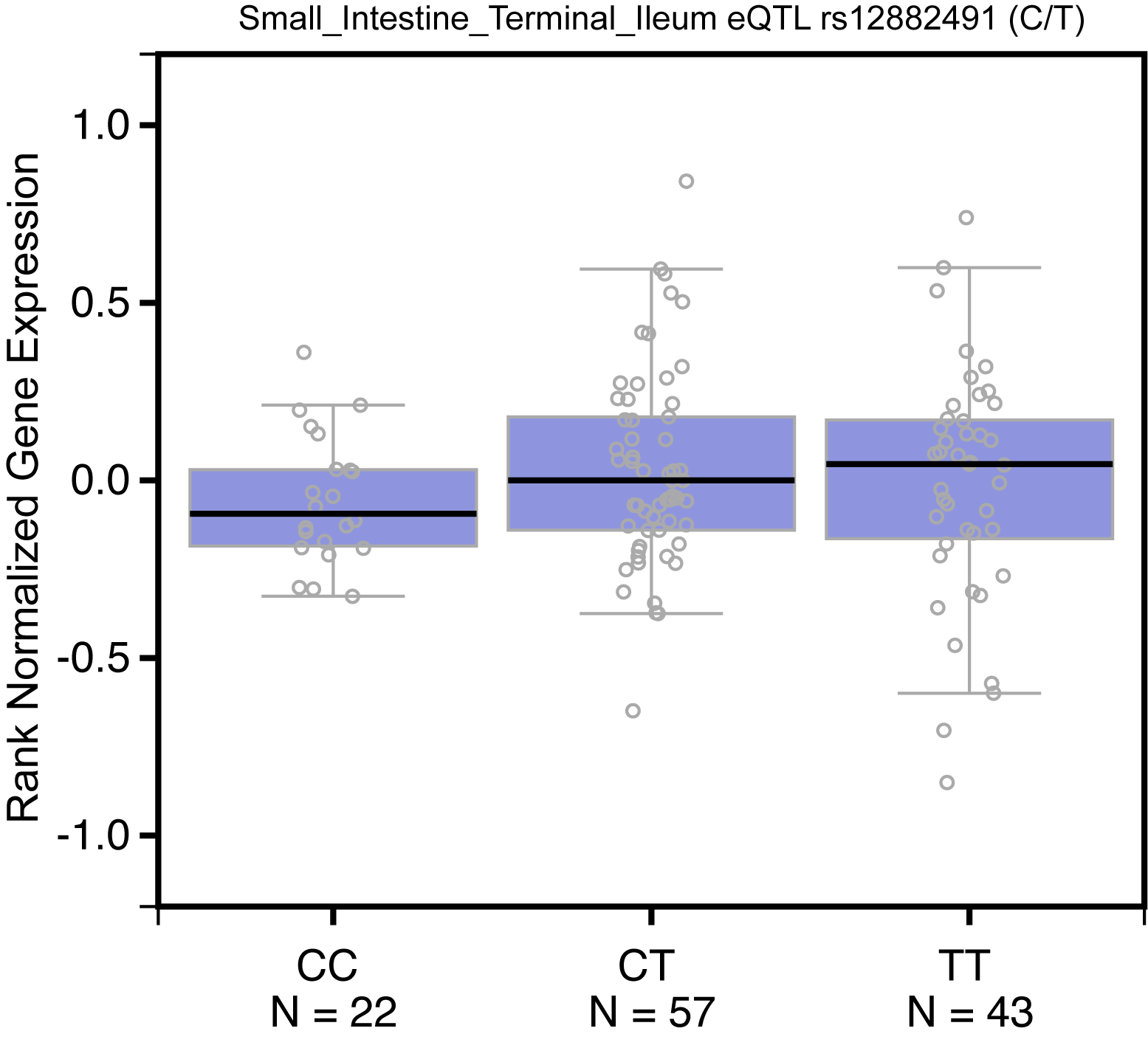
**

**Supplementary Figure 4. GTEx database genotype-gene expression plot for the TRAF3 associated SNP rs12882491 in the small intestine tissue**. Box plots for the different TRAF3 rs12882491 genotypes (C GWAS risk allele) and the rank normalized gene expression levels of the gene for each sample (grey dots). The C allele shows a decreased gene expression pattern compared to the T allele, although, no statistically significant correlation between groups of genotypes is observed.
